# Autosomal Recessive Transmission of a Rare *KRT74* Variant Causes Hair and Nail Ectodermal Dysplasia: Allelism with Dominant Woolly Hair/Hypotrichosis

**DOI:** 10.1371/journal.pone.0093607

**Published:** 2014-04-08

**Authors:** Doroteya Raykova, Joakim Klar, Aysha Azhar, Tahir Naeem Khan, Naveed Altaf Malik, Muhammad Iqbal, Muhammad Tariq, Shahid Mahmood Baig, Niklas Dahl

**Affiliations:** 1 Department of Immunology, Genetics and Pathology, Science for Life Laboratory at Uppsala University, Biomedical Center, Uppsala, Sweden; 2 Human Molecular Genetics Laboratory, Health Biotechnology Division, National Institute for Biotechnology and Genetic Engineering, Faisalabad, Pakistan; 3 Department of Nuclear Medicine, Punjab Institute of Nuclear Medicines Hospital, Faisalabad, Pakistan; Innsbruck Medical University, Austria

## Abstract

Pure hair and nail ectodermal dysplasia (PHNED) comprises a heterogeneous group of rare heritable disorders characterized by brittle hair, hypotrichosis, onychodystrophy and micronychia. Autosomal recessive (AR) PHNED has previously been associated with mutations in either *KRT85* or *HOXC13* on chromosome 12p11.1-q14.3. We investigated a consanguineous Pakistani family with AR PHNED linked to the keratin gene cluster on 12p11.1 but without detectable mutations in *KRT85* and *HOXC13*. Whole exome sequencing of affected individuals revealed homozygosity for a rare c.821T>C variant (p.Phe274Ser) in the *KRT74* gene that segregates AR PHNED in the family. The transition alters the highly conserved Phe274 residue in the coil 1B domain required for long-range dimerization of keratins, suggesting that the mutation compromises the stability of intermediate filaments. Immunohistochemical (IHC) analyses confirmed a strong keratin-74 expression in the nail matrix, the nail bed and the hyponychium of mouse distal digits, as well as in normal human hair follicles. Furthermore, hair follicles and epidermis of an affected family member stained negative for Keratin-74 suggesting a loss of function mechanism mediated by the Phe274Ser substitution. Our observations show for the first time that homozygosity for a *KRT74* missense variant may be associated with AR PHNED. Heterozygous *KRT74* mutations have previously been associated with autosomal dominant woolly hair/hypotrichosis simplex (ADWH). Thus, our findings expand the phenotypic spectrum associated with *KRT74* mutations and imply that a subtype of AR PHNED is allelic with ADWH.

## Introduction

Ectodermal dysplasias (EDs) comprise a heterogeneous group of developmental disorders involving two or more ectodermal appendages [1], [2]. Pure hair and nail ectodermal dysplasia (PHNED (MIM 602032, 614929 and 614931)) is a rare subgroup of EDs characterized by nail dystrophy, brittle hair and hypotrichosis. Both autosomal recessive (AR) and autosomal dominant forms of PHNED have been described, and the clinical expression is highly variable [3]–[5]. Two genes, both of which are located in the type II keratin gene cluster on chromosome 12q12-q14.1, have to date been associated with AR PHNED. *KRT85* mutations were identified in consanguineous families segregating alopecia and nail dystrophy [6], [7] and similar phenotypes were recently associated with mutations in *HOXC13*, a transcription regulator of keratin and keratin-associated protein genes [8], [9], [10]. One additional gene locus associated with AR PHNED has been mapped to the chromosome 17p12-q21.2 region spanning the keratin I gene cluster, but no causative mutation has yet been identified [11].

Hair and nails are designed to resist chemical exposure, thermal variations and mechanical trauma. This protective function is mediated by keratin intermediate filaments built up by a number of keratins, keratin-associated and cross-linked proteins which are expressed in a complex, sequential and tissue-specific manner during development [12]–[15]. More than fifty keratins and keratin-associated proteins contribute to the unique cross-linked heteropolymers of the hair shaft and the nail plate [16]–[18]. Some are detected either in the nails or in the hair shaft but there is also a considerable overlap in the expression pattern supporting shared features for the two appendages.

In the present study, we re-investigated a consanguineous Pakistani family segregating AR PHNED previously linked to chromosome 12p11.1-q14.3 (MIM 614929) [2]. The region coincides with the type II keratin gene cluster and spans both the *KRT85* and the *HOXC13* genes. Analysis of the entire coding region of *KRT85* did not reveal any mutation [2] and we therefore analyzed *HOXC13* and the rest of the approximately 480 linked genes by exome sequencing. The combined results from our study indicate that the *KRT74* gene is a second member of the keratin II gene cluster associated with AR PHNED.

## Methods

### Subjects and Ethics Statement

**Figure 1:**
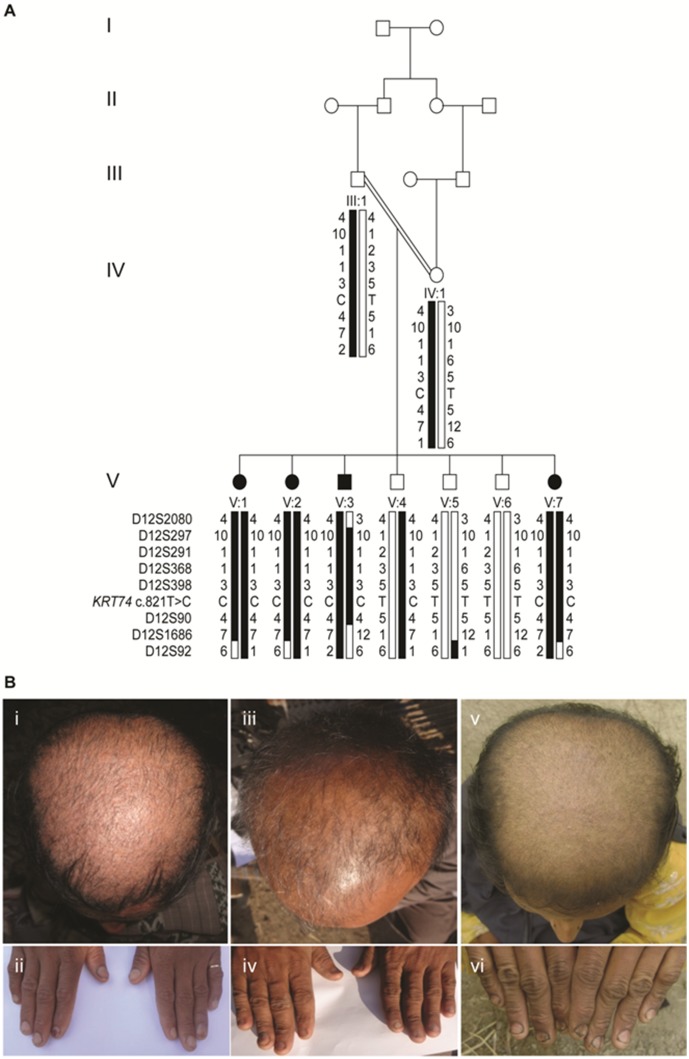
Pedigree of the family segregating AR PHNED and phenotypic features of affected family members. (A) Pedigree of the consanguineous Pakistani family with PHNED (modified from [2]). Affected individuals are represented by filled symbols. Haplotypes are shown below each individual with microsatellite marker alleles on chromosome 12p11.1-q14.3 used for linkage analysis, as well as the relative position of the *KRT74* variant identified. Individuals V:1 and V:3 were selected for exome sequencing. (B) Patients V:2 (panels i, ii), V:3 (panels iii, iv) and V:7 (panels v, vi) show hypotrichosis with brittle hair of the scalp and dystrophic, slightly spoon-shaped nails with distal onycholysis and mild micronychia.

We re-investigated a consanguineous five-generation Pakistani family segregating AR PHNED. The pedigree was slightly modified from the previous report [2] (Figure 1A). Written informed consent was obtained from all participating individuals or their legal guardians and the study was approved by the local ethical committee at the National Institute for Biotechnology and Genetic Engineering (NIBGE), Faisalabad, Pakistan. Animal experiments were approved by the Animal Ethics Committee, Uppsala, Sweden (permit number C 271/12).

### Genetic Analysis

Genomic DNA was extracted from blood using standard methods and whole exome enrichment was performed using the SureSelect v4 50 Mb whole exome kit (Agilent, Santa Clara, CA). After exome capture, the enriched DNA was sequenced using the SOLiD5500xl system (Life Technologies, Carlsbad, CA) and aligned to the human reference genome (hg19). An average of 96% of the exonic baits was covered at least 1x, and 69% were covered by greater than 10x. Common variants were excluded by filtering against the Exome Variant Server (EVS) data (NHLBI GO Exome Sequencing Project, URL: http://evs.gs.washington.edu/EVS/), dbSNP132 (MAF>0.01, URL: http://www.ncbi.nlm.nih.gov/projects/SNP/snp_summary.cgi?build_id=132), as well as against 350 “in house” exomes.

Validation of variants was performed by Sanger sequencing after standard purification, cycle sequencing with a commercial kit (BigDye Terminator v3.1 Cycle Sequencing Kit (Life Technologies, Carlsbad, CA)) and precipitation in HiDi formamide. Segregation analysis was carried out using the BioEdit Sequence Alignment Editor version 7.0.5.3. Primer sequences are available upon request.

### Immunohistochemistry and Immunofluorescent Staining

Formalin-fixed paraffin-embedded skin punch biopsies from healthy human subjects and one affected family member (V:3; Figure 1A), as well as distal digits of adult mice were sectioned and treated with standard methods. In brief, slides were deparaffinized in 100% xylene and rehydrated in decreasing concentrations of ethanol (100% to 80%). Antigen retrieval was performed either by boiling for 8 min, 125°C, 1 atm in a pressure cooker in unmasking solution (Vector Laboratories, Burlingame, CA), or by incubating for 10 minutes with 0.125% trypsin for enzymatic pretreatment (Abcam, Cambridge, UK) at 37°C in a humidified chamber and subsequent washing in 1x PBS.

Immunofluorescent staining was performed as described elsewhere [19]. Briefly, slides were fixed with freshly prepared ice-cold 2% paraformaldehyde and subsequently permeabilized in blocking solution (1x PBS pH 7.4, 1% BSA, 0.1% Triton X 100). Primary guinea pig anti-Keratin-74 antibody (1∶150 (Abcam, Cambridge, UK)) and, when human skin sections were stained, primary rabbit anti-Lamin-B1 antibody (1∶500 (Abcam, Cambridge, UK)) were then allowed to bind overnight at 4°C. After washing the slides in 1x TBS, 0.05% Tween, anti-guinea pig FITC-conjugated IgG (1∶600 (Abcam, Cambridge, UK)) or anti-rabbit AlexaFluor 555 IgG (1∶1000 (Life Technologies, Carlsbad, CA)) was applied for 1.5 hours at room temperature in the dark. Visualization of staining was performed on Zeiss 510 confocal microscope (Carl Zeiss microscopy, Jena, Germany) using Zen 2009 imaging software.

## Results

### Clinical Features

The four affected full siblings (V:1, V:2, V:3 and V:7) presented with sparse and brittle hair of the scalp (Figure 1B, panel i, iii and v), eyebrows and eyelashes. Hair shafts had a shaggy appearance as described previously [2]. Nails of toes and digits were spoon-shaped and dystrophic with distal onycholysis and mild micronychia (Figure 1B, panel ii, iv and vi). Symptoms were present since birth. The affected individuals were otherwise healthy including normal skin, dentition and self-reported normal sweating. The parents (uncle and niece) and three siblings (V:4, V:5 and V:6) showed normal texture of body and scalp hair, as well as normal nails without signs of PHNED. The father (III:1) had abundant scalp hair until approximately 50 years of age and he had a progressive androgen-related alopecia since.

### Genetic Analysis

In light of the recent reports showing AR PHNED mutations in *HOXC13* [10], [20], we first investigated this gene from exome sequencing data obtained from two affected family members (V:1 and V:3, Figure 1A). A coverage gap corresponding to part of exon 1 of *HOXC13* was identified and we therefore analyzed the entire coding region of the *HOXC13* gene in all family members by Sanger sequencing. No sequence variation was observed when compared to the reference sequence (NM_017410.2). Thus, we concluded that *HOXC13* is an unlikely candidate gene, although regulatory and non-coding mutations were not excluded.

**Figure 2:**
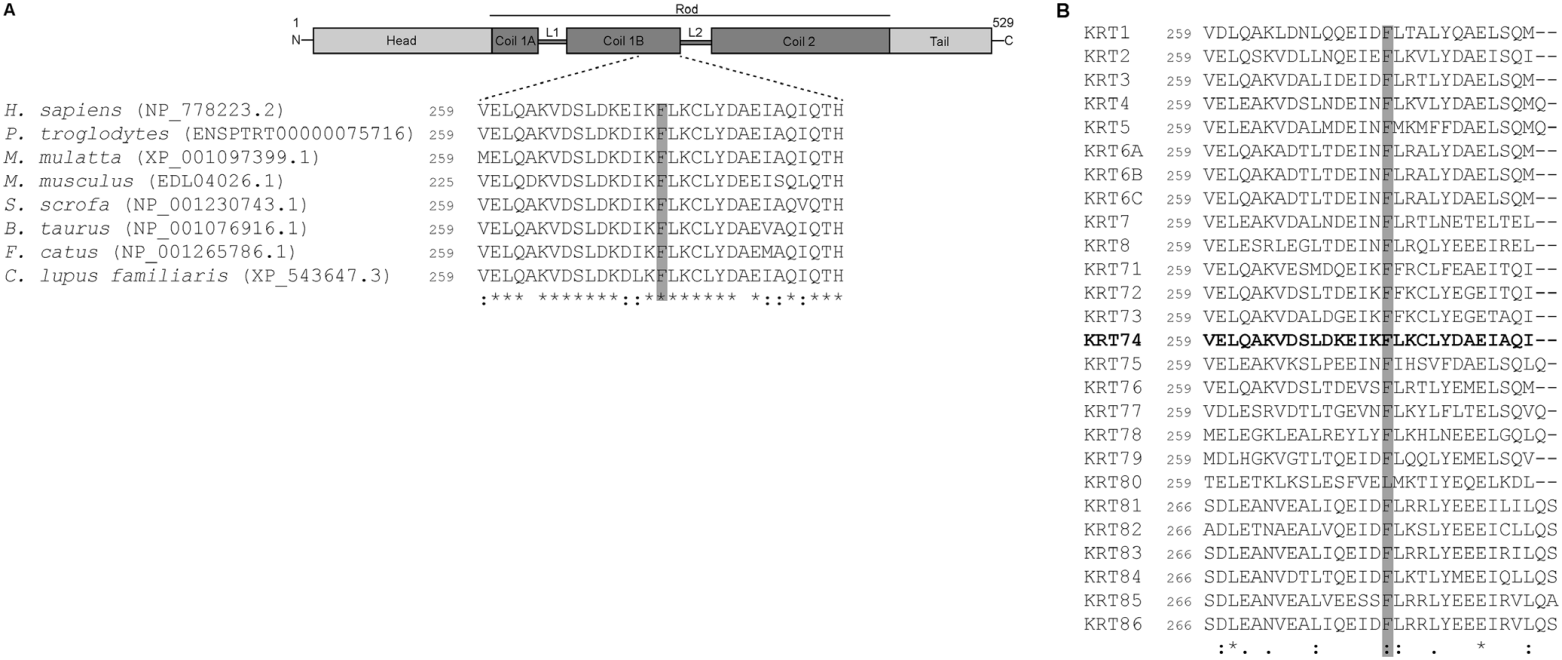
Protein sequences showing inter-species conservation of Keratin-74 and homologies between 26 human type II keratins. (A) Clustal Omega alignment of part of Keratin-74 shows complete evolutionary conservation of the Phe274 residue (shaded) in all species with a known keratin-74 ortholog. (B) Alignment of parts of the 26 human type II keratins illustrating the almost identical homology at amino-acid position 274 (shaded). Only Keratin-80 deviates by having a biochemically similar leucine at this position. Position of the first amino acid shown is indicated to the left of each protein sequence. KRT: Keratin.

Further analysis of the exome sequence data revealed three coding homozygous missense variants in the linked region on chromosome 12p11.1-q14.3. The variants include a c.821T>C transition in *KRT74* (NM_175053; p.Phe274Ser), a c.38C>T transition in *CELA1* (NM_001971; p.Pro13Leu) and a c.1037A>G transversion in *IKZF4* (NM_022465; p.Tyr346Cys). The variant in *KRT74* was predicted as probably damaging (HumVar score 1.00), whereas the variants in *CELA1* and *IKZF4* were predicted as benign (HumVar score 0.01 and 0.10 respectively). The *KRT74* c.821T>C transition is located in exon 4 encoding the coil 1B domain (Uniprot, Q7RTS7) and Clustal Omega analysis showed that the phenylalanine in position 274 of Keratin-74 is completely conserved among all species known to have a Keratin-74 ortholog (Figure 2A). Furthermore, residue Phe274 is conserved in 25 out of the 26 human type II keratins, with the single exception of Keratin-80, in which the phenylalanine is replaced by the biochemically similar leucine (Figure 2B).

Segregation analysis of the *KRT74* variant in the family was consistent with autosomal recessive inheritance. The four affected individuals were homozygous for the *KRT74* c.821T>C variant, whereas the parents and one healthy sibling were heterozygous carriers (Figure 1A, Figure S1). Two healthy brothers were homozygous for the “wild-type” (T) allele.

Analysis of the c.821T>C variant in 350 “in house” exomes, as well as in 200 Swedish and 200 Pakistani control chromosomes turned out negative. Interestingly, the transition is present under entry rs147962513 in the Exome Variant Server (EVS) data (NHLBI GO Exome Sequencing Project, URL: http://evs.gs.washington.edu/EVS/), release of September 2013, with an allele frequency of 0.0002 (2 out of 13006 alleles), as well as in dbSNP132 with an average allele frequency of 0.002. However, none of these databases report the variant in a homozygous state.

### Immunohistochemistry

**Figure 3:**
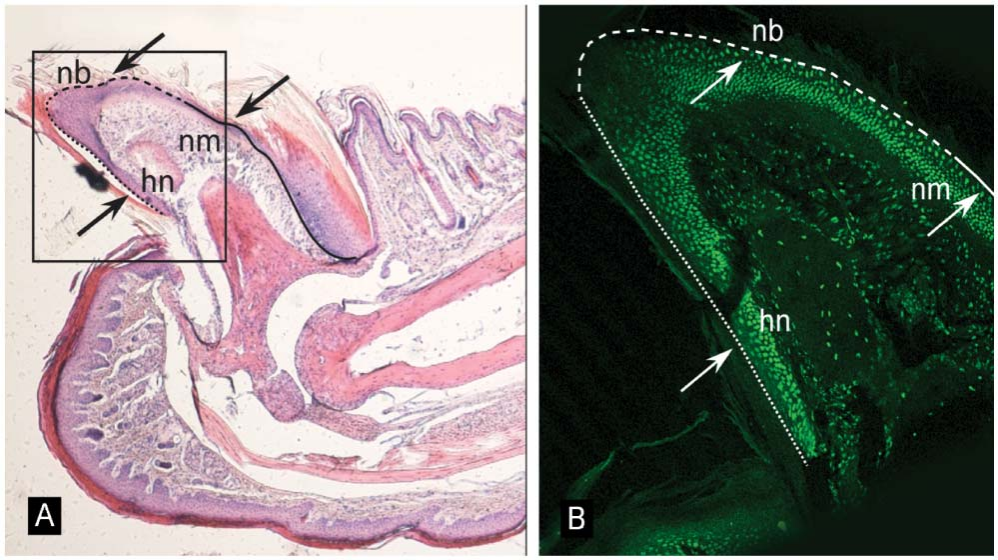
Mouse keratin-74 is expressed in the regenerative nail tissue. (A) H&E staining of a normal mouse nail unit (40x magnification). The continuous line delineates the border of the nail matrix, the dashed line shows the border of the nail bed, and the dotted line illustrates the hyponychium. nb: nail bed, nm: nail matrix, hn: hyponychium. The boxed section is enlarged in (B). (B) Immunofluorescent staining of keratin-74 shows strong expression in the nail matrix, nail bed and hyponychium (100x magnification). Note the single-cell layer immediately adjacent to the nail plate with pronounced staining. The continuous, dashed and dotted lines correspond to the lines presented in (A).

**Figure 4:**
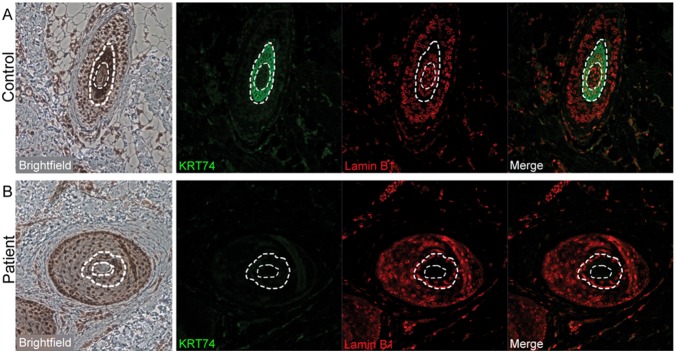
Keratin-74 is not detected in hair follicles of an individual with AR PHNED. (A) Sections from a forearm skin biopsy of a healthy control individual show positive staining for Keratin-74 (KRT74; green) in the inner root sheath of the hair follicle. The nuclear envelope marker Lamin B1 (red) is used for co-staining (200x magnification). (B) Skin biopsy from forearm of individual V:3 with AR PHNED shows a hair follicle that stains negative for Keratin-74. Co-staining with Lamin B1 is normal (200x magnification). Dashed lines delineate the outer border of the inner root sheath, and the inner border of the root sheath with the medulla. From left to right: Brightfield (light) microscopy; Keratin-74 staining (KRT74); Lamin B1 staining and merge.

We further investigated the expression of Keratin-74 in hair follicles from patient and control subjects as well as in regenerating nails using mouse claws. Immunohistochemical staining of claws from distal digits of adult mice showed that keratin-74 is expressed in the epidermis with a particularly strong staining in the nail matrix, nail bed and hyponychium (Figures 3A and 3B). Interestingly, a distinct keratin-74 staining was found in a monolayer of cells in the outer nail bed and just adjacent to the nail-plate (Figure 3B). Immunostaining of sections from human skin punch biopsies of healthy controls showed strong positive staining for Keratin-74 in the inner root sheath of the hair follicle (Figure 4A) and in epidermis (Figure S2) consistent with previous findings [21], [22]. By contrast, epidermis and hair follicles from an affected family member (individual V:3) were negative for Keratin-74 staining (Figure 4B). The analysis was repeated and the results confirmed hair follicles devoid of Keratin-74 in the patient but not in the control subject, whereas co-staining with Lamin B1 was positive in specimens from both individuals (Figures 4A and 4B).

## Discussion

We re-investigated a consanguineous family segregating AR PHNED linked to chromosome 12p11.1-q14.3. Affected family members had congenital hypotrichosis with shaggy hair shafts, spoon-shaped nails, mild micronychia and onycholysis [2]. The clinical expression appeared relatively mild when compared to previously reported forms of AR PHNED, including those associated with *KRT85* and *HOXC13* mutations [2]–[6], [10], [20], [23]–[25]. Sequence variants were previously excluded in the coding regions of *KRT85* [2], and in this study we used exome and Sanger sequencing to exclude mutations in the coding parts of *HOXC13*. Further analysis of exome sequencing data in the chromosome 12p11.1-q14.3 region from two affected individuals revealed three homozygous coding variants, including a *KRT74* missense variant c.821T>C (p.Phe274Ser). All four affected family members were homozygous for the variant, whereas the unaffected parents and three siblings were either heterozygous or homozygous for the wild type allele. We focused our attention on the *KRT74* variant partially because keratins are key components of the cytoskeletal network in epidermis and its appendages. Furthermore, Keratin-74 is expressed in the inner root sheath of hair follicles [21] and in the distal digits during mouse claw development [13].

The missense variant p.Phe274Ser predicts a substitution of the hydrophobic phenylalanine for the polar uncharged serine within the coil 1B domain of Keratin-74. The coil 1B domain is highly conserved among all species with known Keratin-74 sequences and within this domain the Phe274 is completely conserved (Figure 2A) suggesting a functional importance. All members of the keratin family share a common structure [26] including repeats of hydrophobic amino acids in the α-helical rod domain required for dimerization. The α-helix spans the coil 1B domain and contains a motif of 13 residues, the so-called “trigger site”, that provides the interactions necessary for a long-range coil dimerization (Figure 2A) [27]–[30]. In Keratin-5 this trigger site is shown to be of importance for the interaction with Keratin-14 and heterodimer formation [27]–[30]. Keratin-5 and Keratin-74 belong to the same subfamily of “soft” keratins and 66% of their coil 1B domains are identical, including the Phe274 residue. We compared the coil 1B sequence of Keratin-74 to all members of the type II keratins in human. Interestingly, the Phe274 is completely conserved in 25 out of 26 type II human keratins (Figure 2B). Thus, the highly conserved residue 274 in type II keratins strongly argues for its functional importance. Furthermore, knowledge about the function of the coil 1B domain in Keratin-5, with homology to Keratin-74, leads to the hypothesis that the p.Phe274Ser variant in Keratin-74 interferes with hetero- or homodimer formation, which would compromise the stability of intermediate filaments.

Consistent with our clinical and genetic data we confirmed expression of keratin-74 in distal digits of adult mice using immunohistochemistry. The analysis of mouse digits showed a particularly strong staining in the region corresponding to the nail matrix, the nail bed and hyponychium. Interestingly, we observed an ordered monolayer of cells just adjacent to the nail plate with a strong keratin-74 staining, suggesting this protein to be of importance for the interphase of the nail and the nail bed. Furthermore, immunostaining of human skin sections revealed strong expression of Keratin-74 in the inner root sheath of hair follicles in healthy individuals. Interestingly, no expression was detected in hair follicles of an affected family member upon repeated analysis. However, co-staining with the nuclear envelope marker Lamin B1 showed intense expression in both healthy controls and the affected individual. In addition, Keratin-74 staining was strong in epidermis of the control subject but very faint or absent in epidermis of the affected family member. These observations suggest that the p.Phe274Ser variant results in degradation of Keratin-74 and a loss of function. Thus, our combined results from immunostaining strongly support Keratin-74 to be associated with AR PHNED and an important component for the formation and regeneration of both nails and hair.

In addition to the *KRT74* c.821T>C transition, two coding missense variants within the linked region on chromosome 12p11.1 segregate with the disease. These two variants were excluded based on a combination of factors. The c.38C>T (p.Pro13Leu) variant is located in the *CELA1* gene (MIM 130120) encoding a member of the family of serine proteases which hydrolyze elastin, fibrin, and hemoglobin in skin keratinocytes [31], but not in regenerating epidermal layers. According to PolyPhen-2 predictions, the c.38C>T (p.Pro13Leu) variant would not compromise protein function. The second variant c.1037A>G (p.Tyr346Cys) is located in the *IKZF4* gene, a member of the Ikaros family of transcription factors expressed in lymphocytes but not in epidermis. Furthermore, the substitution in *IKZF4* is predicted as benign according to PolyPhen-2.

Heterozygous mutations in *KRT74* have been shown to cause autosomal dominant woolly hair (MIM 194300) [6], [25], and/or hypotrichosis simplex (MIM 613981; collectively ADWH) [25]. These disorders affect texture and regeneration of the hair but without nail involvement, and our findings imply that ADWH and the AR PHNED subtype in our family are allelic conditions. Isolated and tightly curled hair of normal density in ADWH segregates with heterozygosity for the *KRT74* missense variant c.444C >G (p.Asn148Lys) [6], as well as with a splice acceptor site mutation c.IVS8–1G>A [25]. Furthermore, the heterozygous *KRT74* missense variant c.1444G>A (p.Asp482Asn) has been associated with isolated hypotrichosis [25]. In our family, the heterozygous carriers of the c.821T>C (p.Phe274Ser) variant show normal wavy or straight hair with normal density. This observation indicates that the *KRT74* variant c.821T>C is clinically silent in a heterozygous state in contrast to the c.444C>G, c.1444G>A and c.IVS8–1G>A variants. One plausible explanation for the combined hair-nail phenotype associated with a biallelic *KRT74* mutation in our family is a complete loss of Keratin-74 from two mutated alleles. This is supported by the absence of Keratin-74 staining in hair follicles and epidermis of an affected individual. Consequently, heterozygosity for the p.Phe274Ser variant would result in haploinsufficiency for Keratin-74 but still at adequate levels for normal regeneration of hair and nails. This could explain the apparent autosomal recessive inheritance in our family in contrast to most other keratin disorders caused by heterozygous and dominant-negative acting mutations. It is noteworthy that the affected family members show no symptoms from the skin. Keratin-74, normally expressed in epidermis, stained negative for the protein in the patient analyzed. This suggests that Keratin-74 is dispensable for the regeneration of skin in contrast to its essential role for the formation of hair and nails.

Interestingly, the c.821T>C allele has been reported at very low frequency in Exome Variant Server (EVS) and dbSNP132 (2/10 000 and 2/1000, respectively, rs147962513). However, these allele frequencies correspond to an incidence of AR PHNED of no more than one per million and, consequently, would not result in a significant subgroup of patients among the ectodermal dysplasias.

In conclusion, our results from clinical and genetic analysis, functional predictions and immunohistochemistry indicate that homozygosity for the c.821T>C transition causes AR PHNED in our family. The position and the predicted effect of the p.Phe274Ser substitution in the coil 1B domain of Keratin-74 suggest that the mutation interferes with keratin dimerization. The association between the p.Phe274Ser variant and AR PHNED is further supported by the absence of Keratin-74 staining in the hair follicles and epidermis of one patient suggesting the protein to be degraded, possibly as a consequence of absent dimerization. Our findings add to the phenotypic spectrum associated with *KRT74* sequence variants, and further studies are now required to clarify the precise mechanisms mediated by both wild type and mutated Keratin-74 for the formation of keratin intermediate filament complexes of the hair shaft and nail plate.

## Supporting Information

Figure S1 **Chromatograms from Sanger sequencing illustrating the** ***KRT74*** **c.821T>C variant (rs147962513) associated with AR PHNED.** Chromatograms of the unaffected sibling V:6 (top), the heterozygous carrier III:1 without phenotypic manifestations (middle panel) and the affected family member V:1 (bottom). Black arrows indicate the nucleotide position c.821 of *KRT74*.
(TIF)

Figure S2 **Absent Keratin-74 staining in epidermis of an AR PHNED patient.** (A) Sections from a forearm skin biopsy of a healthy control individual show positive staining for Keratin-74 (KRT74; green) in the epidermis. The nuclear envelope marker Lamin B1 (red) is used for co-staining (200x magnification). (B) A forearm skin biopsy of individual V:3 with AR PHNED shows no detectable epidermal expression of Keratin-74. Lamin B1 co-staining appears normal (200x magnification). From left to right: Keratin-74 staining (KRT74); Lamin B1 staining and merge.
(PDF)
