# Supplementary figures and images for: Autosomal Recessive Transmission of a Rare KRT74 Variant Causes Hair and Nail Ectodermal Dysplasia: Allelism with Dominant Woolly Hair/Hypotrichosis
Source: PLoS One. 2014 Apr 8;9(4):e93607. doi: 10.1371/journal.pone.0093607 (PMC3979697; doi:10.1371/journal.pone.0093607)

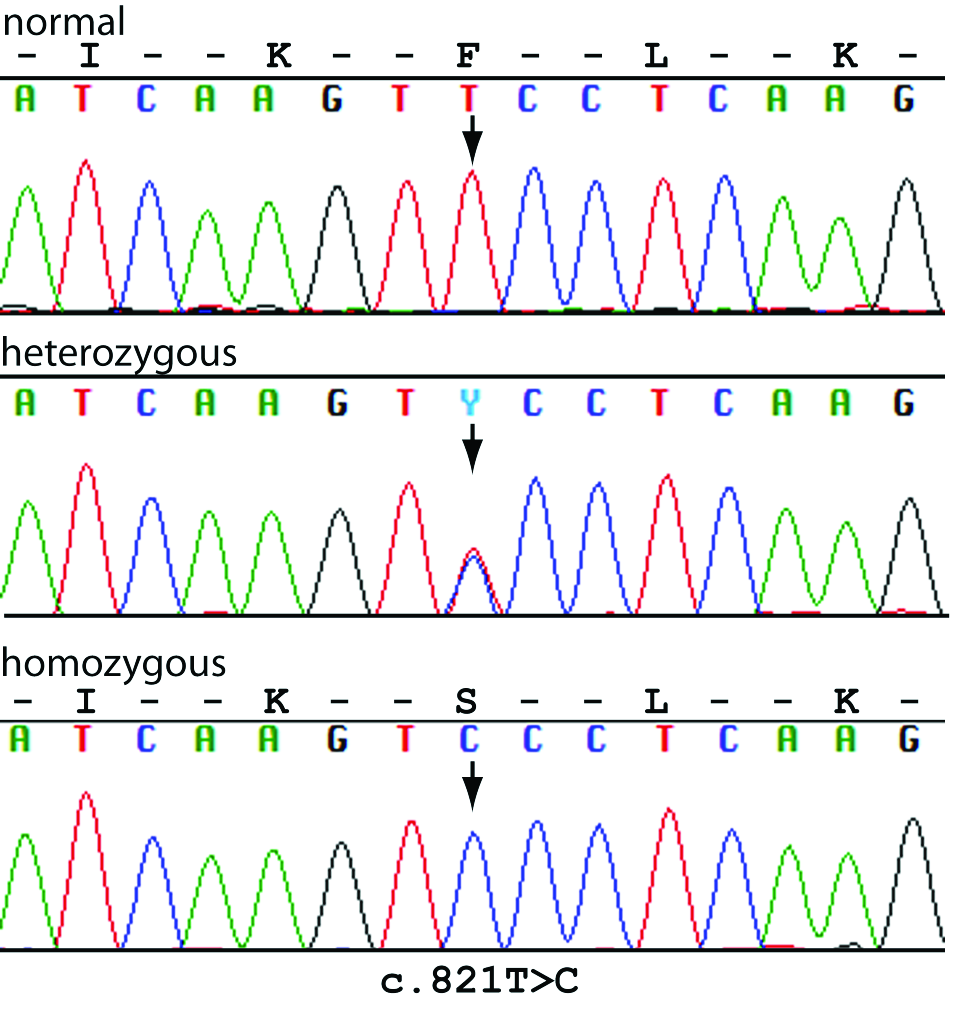

Supplement: Figure S1 — Chromatograms from Sanger sequencing illustrating the KRT74 c.821T>C variant (rs147962513) associated with AR PHNED. Chromatograms of the unaffected sibling V:6 (top), the heterozygous carrier III:1 without phenotypic manifestations (middle panel) and the affected family member V:1 (bottom). Black arrows indicate the nucleotide position c.821 of KRT74. (TIF) [file pone.0093607.s001.tif]

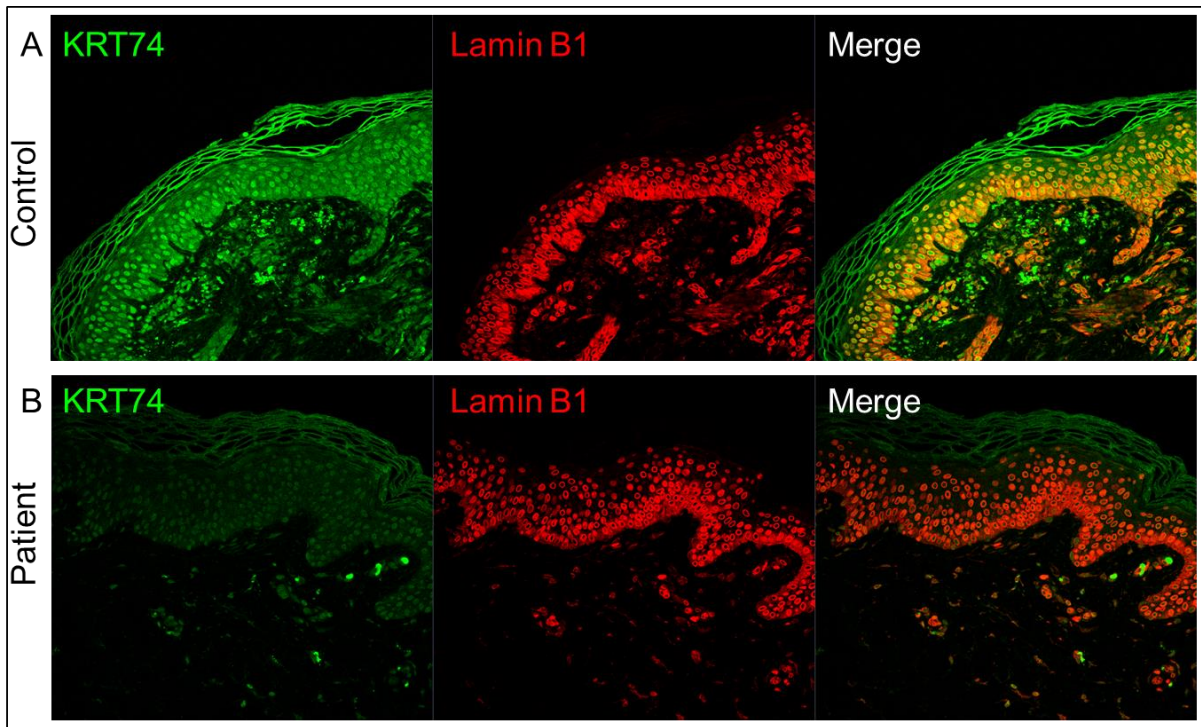

Supplement: Figure S2 — Absent Keratin-74 staining in epidermis of an AR PHNED patient. (A) Sections from a forearm skin biopsy of a healthy control individual show positive staining for Keratin-74 (KRT74; green) in the epidermis. The nuclear envelope marker Lamin B1 (red) is used for co-staining (200x magnification). (B) A forearm skin biopsy of individual V:3 with AR PHNED shows no detectable epidermal expression of Keratin-74. Lamin B1 co-staining appears normal (200x magnification). From left to right: Keratin-74 staining (KRT74); Lamin B1 staining and merge. (PDF) [file pone.0093607.s002.pdf]
